# The Senhance ® assisted laparoscopy in urogynaecology: case report of sacral colpopexy with subtotal hysterectomy with bilateral salpingo-oophorectomy for pelvic organ prolapse ^*^: ^*^ Video Article, to see the video use this link: https://qrco.de/bbdi3G

**Published:** 2020-10-08

**Authors:** G Panico, G Campagna, L Vacca, D Caramazza, S Pizzacalla, V Rumolo, G Scambia, A Ercoli

**Affiliations:** Fondazione Policlinico Universitario A. Gemelli IRCCS, UOC Uroginecologia e Chirurgia Ricostruttiva del Pavimento Pelvico, Dipartimento di Scienze della Salute della Donna e del Bambino e di Sanità Pubblica, Roma, Italia,00168; Università Cattolica del Sacro Cuore, Istituto di Ginecologia ed Ostetricia, Roma, Italia, 00168; 4 PID Ginecologia Oncologica e Chirurgia Ginecologica Miniinvasiva, Università degli studi di Messina, Policlinico G.Martino, Messina, Italia ,98124; Fondazione Policlinico Universitario A. Gemelli IRCCS, UOC di Ginecologia Oncologica, Dipartimento di Scienze della Salute della Donna e del Bambino e di Sanità Pubblica, Roma, Italia, 00168; Department of Gynaecology and Obstetrics, University of East Piedmont “A. Avogadro”, Novara, Italy

**Keywords:** Laparoscopy, Pelvic organ prolapse, Robotic surgery, Sacrocolpopexy

## Abstract

The aim of this case report was to evaluate the feasibility, efficacy, and safety of nerve-sparing laparoscopic sacrocolpopexy (SCP) performed with a minimally invasive approach by using 2.9-mm Senhance ® surgical robotic system (Senhance ® , TRANSENTERIX Inc., USA). A 60-year-old Caucasian woman with symptomatic pelvic organ prolapse-Q (POP-Q) Aa: 2, Ba: 3, C: +4, Bp:2, Ap: 2, TVL:10 underwent subtotal hysterectomy with bilateral salpingo-oophorectomy, with nerve-sparing SCP performed using the Senhance surgical robotic system.. The urogynaecological assessment on the day of discharge and at the 3 month follow-up showed surgical anatomic success (<2 POP-Q stage). The patient was fully satisfied with the cosmetic result. This is the first case of SCP performed with this innovative system. SCP using “Senhance ®” is a feasible and effective approach with good results in terms of operative time, cosmesis, postoperative pain and length of hospitalisation.

## Introduction

Pelvic organ prolapse (POP) is a common condition that causes considerable discomfort and negatively affects the quality of life and daily activities of up to 40% of all women (Handa et al., 2004). It involves the symptomatic descent of the uterus and/or the different vaginal compartments beyond their usual anatomical limits (Haylen et al., 2016). Vaginal bulging, pelvic heaviness, urinary, bowel and sexual dysfunction are the main POP related symptoms (Maher et al., 2016). The lifetime risk of a woman undergoing pelvic organ prolapse (POP)-related surgery is estimated to be 11% (Olsen et al., 1997).

Laparoscopic sacrocolpopexy (LSCP) can be considered the gold standard for treatment of POP, with higher success rates and lower risk of recurrence compared to other techniques (Maher et al., 2016).

Over the last decade the goal of new surgical innovations in minimally invasive surgery (MIS) has been to reduce the invasiveness of the procedures through the reduction of number of the ports or size of instruments without changing surgical techniques and preserving the efficacy and the safety of standard laparoscopy (LPS). The introduction of the first robotic system (da Vinci, Intuitive Surgical System) produced great improvements in terms of the learning curve and feasibility of MIS (Scheib and Fader, 2015). Increased accuracy, enhanced dexterity, faster suturing, and reduced number of errors are the main advantages of Robotic-assisted laparoscopy over conventional laparoscopy. However, there are some specific limitations, such as the absence of tactile feedback and the high costs compared to conventional LPS (Rosero et al., 2013; Pan et al., 2016).

The Senhance ® surgical robotic system (“Senhance ®”, TRANSENTERIX Inc. ,USA) represents an alternative to the traditional robotic system. The new robotic platform consists of a remote- control unit, manipulator arms, and a connection node. The remote 3-dimensional vision, with an eye- tracking camera control system, integrated haptic interaction, and high configuration versatility due to total arm independence, changes the approach to endoscopy procedures. This, combined with the use of 5mm ports and the possibility of having fully reusable tools , demonstrates the strength of this new robotic platform (Rumolo et al., 2019). In this video article we present the feasibility, efficacy, and safety of nerve-sparing LSCP performed with a minimally invasive approach using Senhance ® surgical robotic system.

## Case presentation

A 60-year-old woman who was referred to our Urogynaecology Department at Fondazione Policlinico A. Gemelli for POP, underwent subtotal hysterectomy with bilateral salpingo-oophorectomy with nerve-sparing sacrocolpopexy.

She was Caucasian with body mass index of 28.7 kg/m2) and had symptomatic POP-Q (Aa: 2, Ba: 3, C: +4, Bp:2, Ap: 2, TVL:10).

Pre-operative medical history, physical examination, POP-Q scores evaluation, laboratory exams, and a urodynamic examination were performed. During the pelvic ultrasound evaluation, the uterus and adnexa bilaterally appeared normal. The patient gave a history of two normal vaginal deliveries without complications and no previous surgery. The most relevant symptoms were vaginal bulging and discomfort during sexual intercourse. As she was in menopause, the patient was given the option of undergoing a subtotal hysterectomy and bilateral salpingo-oophorectomy and sacrocolpopexy. She gave informed consent.

## Surgical technique

**Figure 1:**
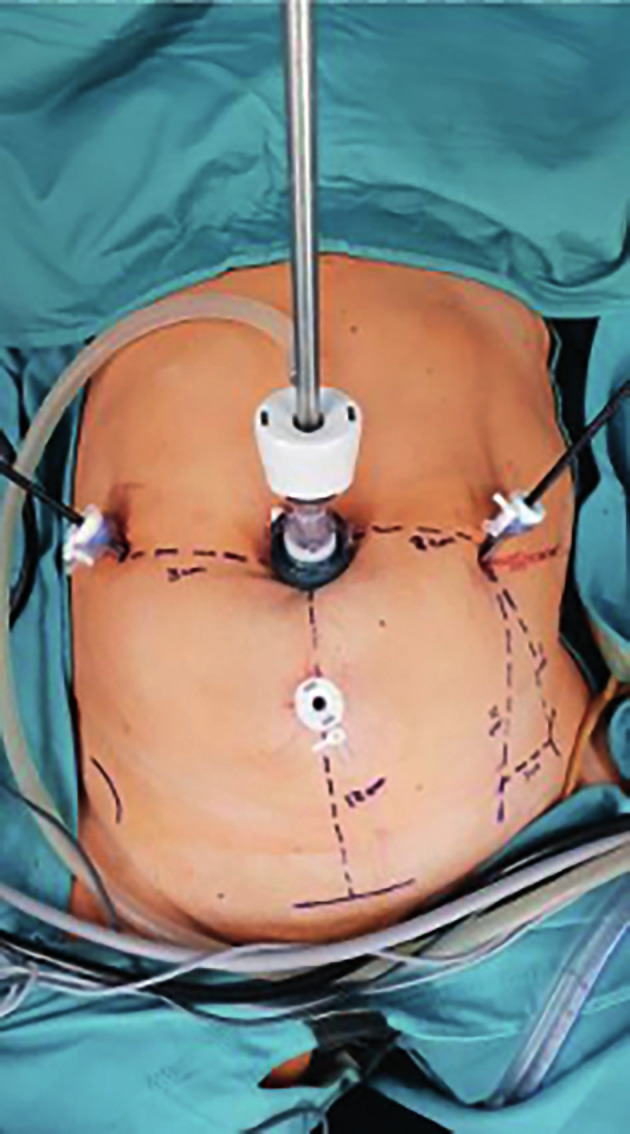
External view of the operating setting.

One transumbilical 10-mm port and three 5-mm ancillary ports were used to perform the surgical procedure. In order to preserve the port setting of conventional laparoscopy, we used three robotic arms; one for the 3D-high-definition 0° 10-mm scope for the intra-abdominal visualisation, and two lateral ports for the operative instruments. A central suprapubic 5-mm port was used for the assisting instruments (suction and irrigation, and grasping) (Figure 1). The robotic fully reusable devices were introduced through the standard ports. Every instrument automatically detected the ideal axis of rotation on the fascia, which became the pivot point of all movements of the instruments. Then, the instruments were connected easily to the robotic arms with magnets, which have the advantage of making the tools easily replaceable. The first surgeon from the cockpit completely controlled the movement of both instruments and optic. The first assistant was situated at the patient’s right side, and the second assistant was in between the legs. The scaled 1:1 force feedback combined with the specially designed handles permitted a safe manipulation of tissue and sutures with the instruments. The first step of LSCP consisted of locating useful anatomic landmarks (outline of the promontory, iliac bifurcation, left common iliac vein, right ureter) and exposing the longitudinal vertebral ligament covering the sacral promontory (https://vimeo.com/429899541/e81f91f2b4). This was accomplished by opening the parietal peritoneum, and gentle sharp and blunt dissection of retroperitoneal tissue. Then the peritoneal incision was extended along the right pelvic side wall up to the uterine isthmus. Subsequently, a subtotal hysterectomy with bilateral salpingo-oophorectomy was performed using the standardised technique in our institution (Gueli Alletti et al., 2018). The peritoneum of pouch of Douglas was incised between the two uterosacral ligaments, and the rectovaginal space was dissected along the posterior vaginal wall. Margins of dissection were the perineal body inferiorly and rectovaginal ligament laterally. An adequately shaped polypropylene mesh (Coloplast Corp, Minneapolis, USA) was placed and fixed to the vaginal wall by four 3-0 non-absorbable sutures. The first two sutures were applied in the midline at the perineal apex of the mesh on the levator ani muscles. Two other sutures were applied, for each side, on the middle and upper portions of the posterolateral vaginal walls at the level of uterosacral ligaments. The vesicouterine peritoneum was opened, and the vesicouterine and vesicovaginal spaces were dissected along the cervical and vaginal walls. Dissection limits were the trigonal region inferiorly and bladder pillars laterally. An adequately shaped polypropylene mesh (Coloplast Corp, Minneapolis, MN55411) was inserted and fixed to the vaginal wall with 3-0 non-absorbable sutures and a non-absorbable barbed suture. The first suture was applied at the midline at the vesical apex of the mesh. Two sutures for each side were placed on the middle and upper portions of anterolateral vaginal walls. The anterior mesh was threaded up toward the promontory under visual control from the vagina so as to lift the prolapsed vaginal walls without excessive tension. The anterior mesh was then fixed to the longitudinal vertebral ligament with one 0 non- absorbable suture on a noncutting needle. After vaginal suspension was obtained, peritonisation was performed, using an absorbable barbed suture. Total operative time was 186 min and 9 min for docking time. The estimated blood loss was 30 mL. No complications were noted according to Dindo classification (Dindo et al., 2004). The patient received antibiotic prophylaxis consisting of cefazoline 2g administered intravenously 1 hour before surgery and antithrombotic prophylaxis consisting of enoxaparin 6000 IU subcutaneously once a day from the day of surgery to the day of discharge. The patient was discharged home on they second postoperative day. Voiding trials began on postoperative day one and drainage was discontinued the same day after resumption of spontaneous and adequate voiding, defined as residual urine volume less than 100 mL on two consecutive postvoid determinations when the volume voided was 200 mL or greater. Pain VAS score decreased after surgery, with a 24 hour value of 3.

The satisfaction value regarding cosmetic outcome was 9/10 for both patients and surgeon.

On the day of discharge, the urogynaecological examination demonstrated a complete resolution of the prolapse. At the three month follow-up the patient confirmed the surgical anatomic success (<2 POP-Q stage) and the degree of overall satisfaction of the cosmetic results was confirmed by the surgeon and the patient equally, with a VAS value of 9/10. Symptoms of prolapse had disappeared completely.

## Discussion

The published literature suggests that robotic surgery should not replace conventional laparoscopic or vaginal procedures but has to be considered as an alternative minimally invasive surgery approach. In fact, surgeons who are not skillful in standard LPS may offer their patients a MIS approach using robotic LPS (Kim et al., 2008). The robotic approach overcomes the drawbacks of laparoscopy, providing a high quality three-dimensional visualiszation, a favourable ergonomy, and allowing more freedom of movement , thus allowing precise and comfortable dissection, maintaining superimposable effectiveness and cure rates (Panico et al., 2020;Smith and Raz, 2009;[Bibr B17][Bibr B18]).

More specifically the “Senhance ®” system combines aspects of laparoscopic and robotic surgery, integrating the advantages of both techniques.

Unlike other robotic platforms, in the “Senhance ®” the operator has haptic and tactile feedback, and each robotic arm is independent of the others, hence allowing for their positioning anywhere in the surgical field through a standard 5-mm trocar located in the same position used during standard laparoscopy.

In addition, the trocar size is lower than that of the Da Vinci (5mm vs 8mm). It is thus straightforward to understand why this system gives the surgeon the possibility of opting for an easy and fast conversion to a standard laparoscopic setting in case of need, also considering that it guarantees better cosmetic outcomes compared with those of other robotic platforms (Noor et al., 2015).

The operative time (OT) for the surgical treatment was longer compared to standard laparoscopy, but similar to those described for robot assisted laparoscopy (Pan et al., 2016). The operative time requiredfor the supracervical hysterectomy and bilateral salpingo-ophorectomy may have affected the overall durationalthough no specific difficulties were encountered in our case. In contrast, the absence of uterine corpus might have been helpful during the mesh positioning and peritonisation step. Some authors reported an increased risk of reoperation after uterine preserving surgery for POP due to the increased risk of cervix elongation (Meritxell et al., 2015), and better functional results from concomitant subtotal hysterectomy (rather than hysteropexy), due to preservation of pericervical ring (Meritxell et al., 2015;Saliba et al., 2019). Additionally published data clearly show an age-related risk of uterine corpus and ovarian cancer (but not for cervical cancer) which exponentially increases in perimenopausal and menopausal age (Memon, 2009). For these reasons we believe supracervical hysterectomy (with bilateral salpingectomy or salpingo-oophorectomy according to age and patient preference) could be offered to perimenopausal and menopausal women, without reproductive desire, after appropriate counselling. One of the factors that influenced the operative time was the need to relocate the robotic arms when a limiting position was reached.

In the Senhance ® system the first surgeon has to use the lateral operative arms during the procedure, different from standard LPS which involves using the lateral and central ports. This can represent a limitation in some cases of large uterus removal when a retroperitoneal pelvic dissection is needed requiring a robotic arm relocation to avoid limited motion.

The Senhance ® system is continuing to evolve. An example of this is the project of introducing new a multifunctional energy device and 3mm tools that can make procedures faster and less invasive.

This is the first case of SCP performed with this innovative system.

In conclusion, SCP performed using the Senhance ® system is a feasible and effective approach with good results in terms of operative time, cosmetic results, postoperative pain and length of hospitalisation.

Large series are mandatory to define the benefits, advantages, and costs of this novel approach.

## Video scan (read QR)

https://qrco.de/bbdi3G

**Figure:**
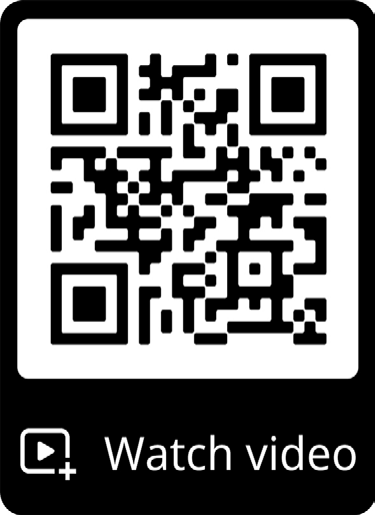


## References

[1] Dindo D, Demartines N, Clavien PA (2004). Classification of surgical complications: a new proposal with evaluation in a cohort of 6336 patients and results of a survey.. Ann Surg.

[2] Gueli Alletti S, Rossitto C, Cianci S (2018). The SenhanceTM surgical robotic system (‘Senhance’) for total hysterectomy in obese patients: a pilot study.. J Robot Surg.

[3] Handa VL, Garrett E, Hendrix S (2004). Progression and remission of pelvic organ prolapse: a longitudinal study of menopausal women.. Am J Obstet Gynecol.

[4] Haylen B, Maher CF, Barber MD (2016). An International Urogynecological Association (IUGA) / International Continence Society (ICS) Joint Report on the terminology for female pelvic organ prolapse (POP).. Int Urogynecol J.

[5] Kim YT, Kim SW, Jung YW (2008). Robotic surgery in gynecologic field.. Yonsei Med J.

[6] Maher C, Feiner B, Baessler K (2016). Surgery for women with apical vaginal prolapse.. Cochrane Database Syst Rev.

[7] Memon A Epidemiology of gynaecological cancers In Shafi M Earl H Tan L (Eds.) Gynaecological Oncology. Cambridge: Cambridge University Press 1-14

[8] Meritxell G, Maria P, Eduardo B Comparison between laparoscopic sacral hysteropexy and subtotal hysterectomy plus cervicopexy in pelvic organ prolapse: a pilot study. Neurourol Urodyn.

[9] Noor N, Rahimi S, Pereira E (2015). Patient preferences for abdominal incisions used for pelvic organ prolapse surgery.. Female Pelvic Med Reconstr Surg.

[10] Olsen AL, Smith VJ, Bergstrom JO (1997). Epidemiology of surgically managed pelvic organ prolapse and urinary incontinence.. Obstet Gynecol.

[11] Pan K, Zhang Y, Wang Y (2016). A systematic review and meta-analysis of conventional laparoscopic sacrocolpopexy versus robot-assisted laparoscopic sacrocolpopexy.. Int J Gynecol Obstet.

[12] Panico G, Giuseppe C, Vacca L (2020). Minimally invasive surgery in urogynecology: a comparison of standard laparoscopic, minilaparoscopic, percutaneous surgical system and robotic sacral colpopexy. Minerva Med.

[13] Rosero EB, Kho KA, Joshi GP (2013). Comparison of robotic and laparoscopic hysterectomy for benign gynecologic disease.. Obstet Gynecol.

[14] Rumolo V, Rosati A, Tropea A (2019). Senhance robotic platform for gynecologic surgery: a review of literature.. Updates Surg.

[15] Saliba E, Nisolle M, Tchente C (2019). Gynecol Obstet Fertil Senol. Do we need to perform systematic supracervical hysterectomy during laparoscopic sacrocolpopexy.

[16] Scheib SA, Fader AN (2015). Gynecologic robotic laparoendoscopic single-site surgery: prospective analysis of feasibility, safety, and technique.. Am J Obstet Gynecol.

[B17] Serati M, Bogani G, Sorice P (2014). Robot-assisted sacrocolpopexy for pelvic organ prolapse: a systematic review and meta-analysis of comparative studies.. Eur Urol.

[B18] Seror J, Yates DR, Seringe E (2012). Prospective comparison of short-term functional outcomes obtained after pure laparoscopic and robot-assisted laparoscopic sacrocolpopexy.. World J Urol.

[19] Smith AL, Raz S (2010). Current status of robotic surgery for pelvic organ prolapse.. BJU Int.

